# In-Memory Logic Operations and Neuromorphic Computing in Non-Volatile Random Access Memory

**DOI:** 10.3390/ma13163532

**Published:** 2020-08-10

**Authors:** Qiao-Feng Ou, Bang-Shu Xiong, Lei Yu, Jing Wen, Lei Wang, Yi Tong

**Affiliations:** 1 School of Information Engineering, Nanchang Hangkong University, Nanchang 330063, China; Ou.Qiaofeng@nchu.edu.cn (Q.-F.O.); xiongbs@nchu.edu.cn (B.-S.X.); yulei@nchu.edu.cn (L.Y.); wenj@nchu.edu.cn (J.W.); 2 College of Electronic and Optical Engineering & College of Microelectronics, Nanjing University of Posts and Telecommunications, Nanjing 210023, China

**Keywords:** in-memory logic operation, non-volatile, random access memory, von Neumann bottleneck, neuromorphic computation

## Abstract

Recent progress in the development of artificial intelligence technologies, aided by deep learning algorithms, has led to an unprecedented revolution in neuromorphic circuits, bringing us ever closer to brain-like computers. However, the vast majority of advanced algorithms still have to run on conventional computers. Thus, their capacities are limited by what is known as the von-Neumann bottleneck, where the central processing unit for data computation and the main memory for data storage are separated. Emerging forms of non-volatile random access memory, such as ferroelectric random access memory, phase-change random access memory, magnetic random access memory, and resistive random access memory, are widely considered to offer the best prospect of circumventing the von-Neumann bottleneck. This is due to their ability to merge storage and computational operations, such as Boolean logic. This paper reviews the most common kinds of non-volatile random access memory and their physical principles, together with their relative pros and cons when compared with conventional CMOS-based circuits (Complementary Metal Oxide Semiconductor). Their potential application to Boolean logic computation is then considered in terms of their working mechanism, circuit design and performance metrics. The paper concludes by envisaging the prospects offered by non-volatile devices for future brain-inspired and neuromorphic computation.

## 1. Introduction

**Figure 1:**
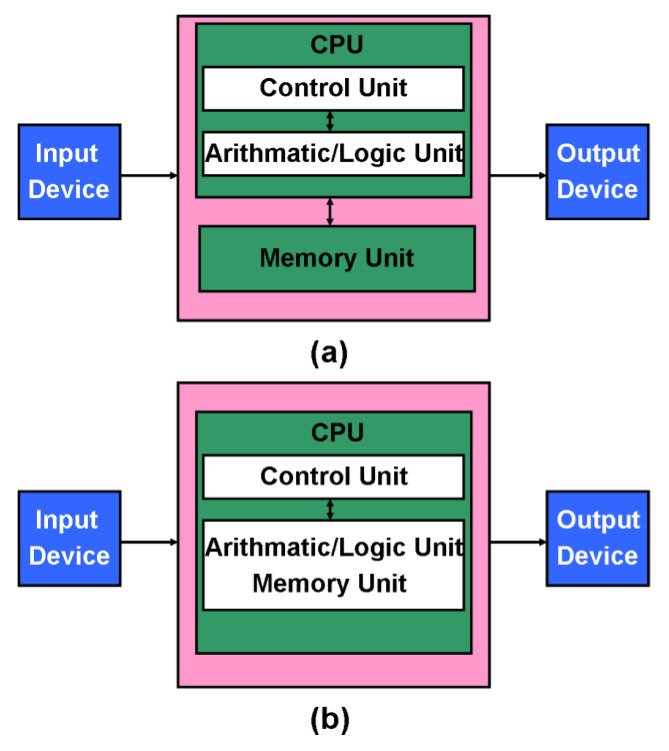
Computer architectures with (**a**) a von Neumann structure and (**b**) a non-von Neumann structure. Note that data need to be retrieved from conventional memory first and transferred to the central processing unit (CPU) for computation when using a von Neumann structure. For non-von Neumann structures, the data can be stored and executed simultaneously inside the computational memory.

The ultimate dream for many computer scientists is being able to create a brain-like computer that can think, determine, and reason like human beings. The rapid recent development of deep neural networks (DNNs) [1,2], in conjunction with advanced computer configurations, such as low-power graphical processing units (GPUs) and fast central processing units (CPUs), has brought this dream much closer to reality. This has been demonstrated by the IBM Watson’s victory (International Business Machines Corporation) over two former world champions in Jeopardy [3] and the AlphaGo beating an 18-times human world Go champion [4]. Although current artificial intelligence (AI)-based machines exhibit greater computational capabilities than human beings, their energy requirements are orders of magnitude higher than that of the human brain. This can be attributed to what is known as the von Neumann bottleneck [5]. This arises from the fact that data are executed and stored in two completely different places in conventional computers, namely the CPU and the main memory. As a result of the von Neumann bottleneck, the CPU has to retrieve data from memory prior to processing it, then transfer it back to memory at the end of the computation. This leads to extra energy consumption during the data transfer process, diminishing the energy efficiency of computational devices [6]. This is aggravated by the fact that the transfer rate of the data bus that connects the CPU with the memory is much slower than that of the CPU, seriously restricting the processing speed [7]. In view of this issue, developing a novel computer architecture that can overcome the von Neumann bottleneck is imperative for the realization of effective brain-like computers (see Figure 1). The key here is to find a way of having all the computation happen uniquely within the memory, so that the shuttling of data to and from is no longer necessary. Approaches adopting this kind of solution are known as ‘in-memory computing’ [8].

**Figure 2:**
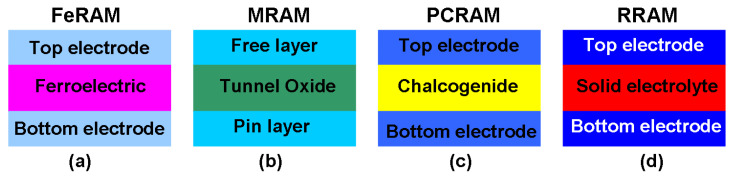
Layered structure of the principal non-volatile RAMs (random access memories): (**a**) FeRAM (ferroelectric RAM); (**b**) MRAM (magnetic RAM); (**c**) PCRAM (phase-change RAM) and (**d**) RRAM (resistive RAM).

In-memory logic operations relate to where the memory is not just used for data storage, but where different physical states of the materials making up the memory are themselves able to represent logical states and operations. Certain kinds of technology offer the scope to realize this possibility, notably non-volatile RAM. Recent progress in the development of non-volatile nanoscale random access memories (RAMs) has led to them offering a viable solution to the von Neumann bottleneck. These devices come mainly in the form of ferroelectric RAM (FeRAM) [9,10,11,12,13], magnetic RAM (MRAM) [14,15,16,17,18], phase-change RAM (PCRAM) [19,20,21,22,23], and resistive RAM (RRAM) [24,25,26,27,28] (see Figure 2). They usually have two distinct physical states relating to when they are either immune to or subject to external excitations. This can be denoted by the binary codes ‘0’’ and ‘1’. Critically, these proposed RAMs can be rapidly and reversibly switched between their different states, thus offering the possibility of realizing in-memory logic operations, which is considered the key CPU requirement in nanoscale devices. Non-volatile RAM offers a number of key advantages in this regard, including its high integration density [29,30,31,32], fast switching speed [33,34,35,36], low energy consumption [37,38,39,40], and long data retention [41,42,43,44]. This makes them eminently suitable for the development of future computational memory applications. However, to date, there has not been a comprehensive description of the physical mechanisms associated with how these promising devices might perform computational tasks, especially logic operations, in the memory itself. Nor has there been any systematic exploration of how in-memory logic operations might be applied and the benefits that might accrue to using non-volatile memory (NVM)-based in-memory logic as opposed to conventional ways of handling logic operations.

This paper is focused on the development of various non-volatile devices where storage and logic computation can be processed together, also known as in-memory computing devices. In-memory computing devices are those that take advantage of their physical state to store data or to provide input/output signals, rather than relying upon electrical charge [8,45]. The paper particularly seeks to provide in a single place a thorough review of the scope of current developments in the use of such devices as a resource for the rapid processing of logic operations. The paper is divided up into four main sections, the first concerning FeRAM, the second MRAM, the third PCRAM, and the fourth RRAM. Each main section begins by describing the basic physical principles of the approach. This is followed by consideration of how the approach can be used for the processing of logic operations. Each section concludes with an outline of the technology’s principle advantages and disadvantages, remaining challenges and recent developments. The structure of each independent section therefore provides an analytic, evaluative and comparative review of the state-of-the-art for each of the primary non-volatile RAM technologies. The paper concludes with a comparative assessment of the pros and cons of each approach for handling logic operations and the prospects offered by these remarkable devices for the future development of computational memory. The paper’s overall goal is to help researchers to better understand the potential of non-volatile RAM and to hopefully further trigger innovations in the field.

## 2. Logic in Ferroelectric RAM

### 2.1. The Technology

**Figure 3:**
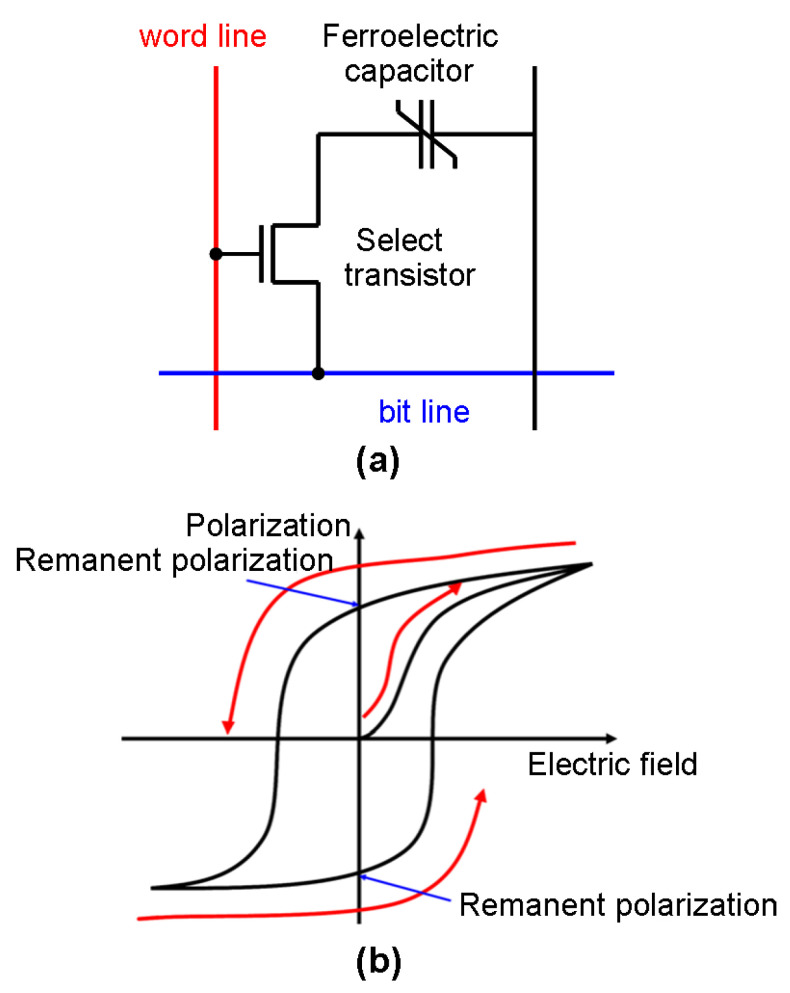
FeRAM structure and polarization: (**a**) a 1T1C-based structure (FeRAM storage unit); (**b**) a ferroelectric loop.

The storage and computational functionality of Ferroelectric RAM (FeRAM) strongly relies upon a ferroelectric material whose polarization is controlled by an external electric field and its stored charge. The simplest FeRAM storage unit comprises a capacitor and a transistor (i.e., a 1T1C structure, see (Figure 3a) [46]. Observations show that the applied field can align the magnetic dipole in the ferroelectric material with the field direction due to shifts in the position of atoms and the distribution of the electronic charge in the crystalline structure. Removing the external field returns the dipole to its original position. Figure 3b shows how the dependence of the polarization on the applied field results in a hysteretic loop. The two potential polarizations in a positive or negative direction can indicate the binary bits ‘0’ and ‘1’, respectively. Thus, FeRAM-based recording can be accomplished by charging the capacitor through the external electric field to switch the dipoles between a positive and negative direction. Unlike other RAM, FeRAM makes use of a peculiar readout mechanism by consistently maintaining the storage cell in a ‘0’ state [10,47]. This simply indicates that no action (i.e., no readout signal) occurs when the cell is held in its original ‘0’ state. To generate a ‘1’ state, polarization re-orientation is required to switch the cell back to its ‘0’ state. The current pulse resulting from this polarization switching is considered to be the readout signal. This readout mechanism is also known as ‘destructive readout’, because it requires the overwriting of the data prior to reading, with the data being rewritten back to the cell after being read.

### 2.2. Potential for Logic Operations

**Figure 4:**
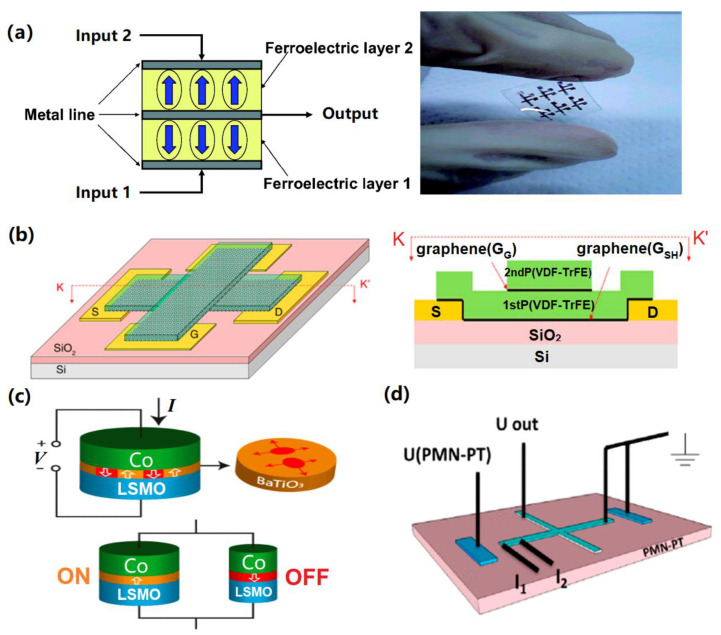
More advanced approaches to FeRAM devices: (**a**) the cross-sectional geometry of an FePLG (ferroelectric programmable logic gate) (left) and a photograph of an FePLG fabricated on a flexible substrate (right); (**b**) the three-dimensional geometry of a graphene field-effect transistor (left) and its cross-sectional view (right); (**c**) the structure of a ferroelectric tunnel memristor; (**d**) a schematic of the device structure for logic gates. ((**a**) is reprinted with permission from [50]; (**b**) is reprinted with permission from [51]; (**c**) is reprinted with permission from [52]; (**d**) is reprinted with permission from [53]).

The use of FeRAM for logic operations can be traced back to 2004, when a non-volatile functional pass gate that could read out the polarization for the on/off state of the pass gate transistor was developed [48]. This was able to adhere to a conventional Boolean logic truth table, thus achieving a sort of ‘in memory’ logic computation and was subsequently expanded to a non-volatile programmable logic device (NVPLD) with a configuration block, a PLD block and a control block. SPICE (Simulation Program with Integrated Circuit Emphasis) simulations demonstrated the feasibility of using NVPLDs at a low cost, operational voltage and power consumption for rapidly reconfigurable and secure field programmable systems [49]. However, despite these achievements, FeRAM largely serves as a switch in these devices, with the critical logic function still being controlled by conventional transistors and D flip-flop circuits. This is space-hungry and can impair the integration density of the FeRAM. Recent advances in semiconductor manufacturing have led to the possibility of FeRAM-based logic memories that are less dependent on CMOS transistors. One idea is to replace inorganic ferroelectric materials with an organic counterpart, such as vinylidene fluoride (VDF). This can be used for a novel ferroelectric programmable logic gate (FePLG) by stacking two ferroelectric capacitors [50] (see Figure 4a). A FePLG has two inputs and one output, with the output value, or switched signal, depending on the input values and the polarization direction in the ferroelectric layers. Applying +Vs/−Vs and −Vs/+Vs to inputs 1 and 2, respectively, results in an output value of +Q, due to the reversed polarization of the first layer. Applying +Vs to both inputs leads to an output value of +2Q because of the reversed polarization of both layers. By choosing different output threshold values, multiple logic functions such as ‘AND’ and ‘OR’ can be achieved. This methodology led to the development of another logic-gate device, where three distinct graphene-ferroelectric hybrid ribbons (GFeR) are stacked (Figure 4b) [51]. A ferroelectric film in the first and second GFeR acts as a gate dielectric, while the graphene in the second GFeR behaves like the common channel in two vertically stacked field-effect transistors. However, the graphene layers in the first and third GFeRs act as gate electrodes, with the ferroelectric film in the third GFeR wrapping the gate graphene. To enable Boolean logic here, positive/negative pulses applied to the gate electrodes represent the logical states, ‘1’/’0’, while the channel resistance indicates the output of the logic gate. In this design, the p-type graphene becomes very depleted and causes a high-resistance state when applying positive pulses to both electrodes. Applying negative pulses to both electrodes results in a low output resistance. Carefully choosing a reference resistance allows for the realization of fundamental logic functions such as ‘OR’ and ‘AND’. In the proposed design, more than one cell was adopted to conduct the Boolean function. This complicates the device architecture.

Recently, a novel ferroelectric tunnel memristor (FTM)-in-memory approach has been proposed where logic operations are processed inside a single memristor [52]. The proposed FTM (see Figure 4c) has a ferroelectric ultrathin barrier (BaTiO_3_) sandwiched between two different electrodes (Co and La_0.67_Sr_0.33_MnO_3_). The tunnel resistance is considered to be the output signal and can be switched between ‘ON’ and ‘OFF’ states, depending on the direction of a spontaneous polarization controlled by an external voltage. Here, the logic block connects an FTM to a loading resistor, giving three terminals. The amplitude of the input voltages between two pairs of the terminals is used to denote the logical states ‘1’ and ‘0’. The FTM is programmed to a specific resistance state by varying the amplitude and duration of the input pulses. A small reading pulse is then applied to another pair of terminals to read the FTM resistance. A high resistance is only programmed in if both pulses are of a large amplitude. A low resistance arises if at least one input pulse is of a small amplitude, corresponding to the ‘NAND’ function. 

Even more recently, a hybrid ferromagnetic-ferroelectric device has been devised, with ferromagnetic/heavy metal films on a ferroelectric (1-x)[Pb(Mg_1/3_Nb_2/3_)O_3_]-x[PbTiO_3_] (PMN-PT) substrate. This allows for deterministic switching from the virgin state of the PMN-PT by using small voltages [53]. The voltages applied to the current channel and the PMN-PT electrodes are defined as the inputs, with the Hall resistance signals being the outputs. A positive and zero Hall resistance represents ‘1’ and ‘0’, respectively (Figure 4d). By tailoring the polarity and magnitude of the input pulses, different logic functions, including ‘XNOR’, ‘AND’, ‘NAND’, and ‘NOT’, have been successfully realized. This spin logic scheme was reported to be simple, scalable, and programmable, with a low energy consumption.

### 2.3. Comparative Advantages and Disadvantages

Compared to its compatriots, FeRAM has a number of merits: low power consumption; a fast writing speed at potentially ns levels; and endurance of up to 10^13^ cycles [54]. Its main disadvantage is its lack of scalability difficulty. This is because ferroelectric materials usually lose their ferroelectric characteristic when they are very thin [55]. Research has therefore been devoted to finding new ferroelectric materials and to developing novel device architectures. In relation to potential new materials, metal oxide-doped HfO_x_ has recently received considerable attention, due to its ability to provide high integration density in 28 nm nodes [56]. It also has a fast switching speed of 20 ns and a long endurance of 10^9^ cycles [57].

An alternative approach to increasing the areal density of FeRAM is to replace the capacitor-type FeRAM with a Fe field-effect transistor (FeFET). This significantly reduces the physical thickness of the gate stack, thus improving the scalability [58,59,60]. A number of potential advantages of FeFETs have been noted in the literature, including improved switching, a number of possible operating modes, and, of particular interest for this paper, the possibility of demonstrating analog synaptic behavior [61]. The main problems associated with FeFET relate to its rewrite capacity and retention time, both of which can be mitigated by using gate-stacked layers of SrBi_2_Ta_2_O_9_ (SBT)/HfNO_2_ (paraelectric layers) [60]. In addition to FeFET, ferroelectric tunnel junction (FTJ)-based devices constitute another promising use of FeRAM. Here, a thin ferroelectric layer is sandwiched between two metal electrodes [62]. The tunnel current across the ferroelectric layer can be modulated via the polarization of the ferroelectric layer, so, FTJ-based devices present the possibility of having a non-destructive readout mechanism [12,63]. Using HfO_2_ as the thin ferroelectric material in an FTJ memory can reportedly give rise to a large on-off tunnel current ratio [54].

The main challenges confronting FeRAM relate to the need to develop new configurations of ferroelectric materials that can effectively solve the existing rewrite and retention issues in a scalable fashion.

## 3. Logic in Magnetic RAM

### 3.1. The Technology

**Figure 5:**
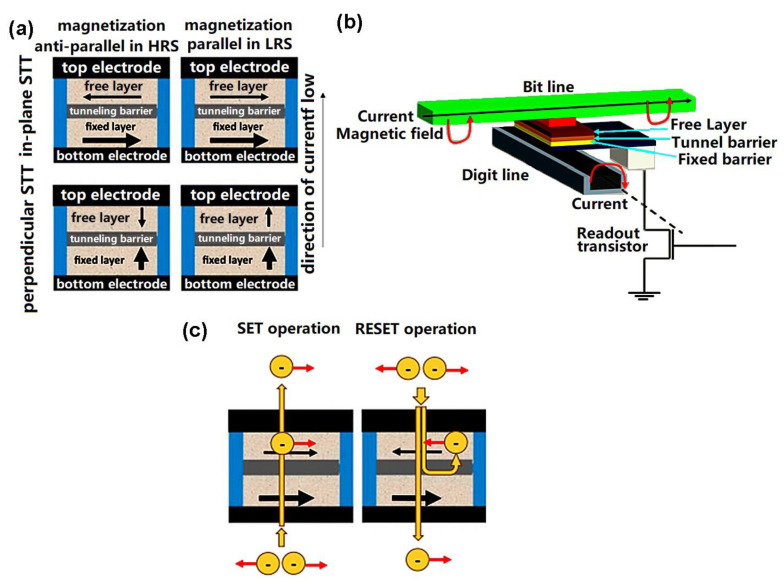
MTJ (magnetic-tunnel junction) and MRAM structure and operation: (**a**) the structure of an MTJ with a tunneling barrier sandwiched between a free layer and a fixed layer; (**b**) the cell architecture of a typical MRAM; (**c**) the ‘set’ (i.e., write) and ‘reset’ (i.e., re-write) operations of MRAM, according to the relative orientation of the magnetization direction of the fixed and free magnetic layers. ((**a**,**c**) are reprinted with permission from [64] and (**b**) is reprinted with permission from [47].)

Magnetic RAM is able to provide memory behavior as a result of its tunnel magneto-resistance (TMR), which usually takes place in a magnetic-tunnel junction (MTJ). This consists of a thin insulator sandwiched between two ferromagnets (see Figure 5). As with FeRAM, by applying an external magnetic field the magnetization direction of the two ferromagnets can be switched. 

If the magnetization direction is parallel, the electrons can more easily tunnel across the insulating layer, leading to a state of low resistance. Opposing magnetization directions generally produce a high-resistance state. Thus, an MTJ can be switched between a high-resistance state and a low-resistance state according to the orientation of the magnetization [64]. Today, thermal-assisted switching (TAS) [65,66,67] and spin transfer torque (STT) switching [68,69,70] are generally considered to be the best approaches to controlling the current. The basic physical principles associated with TAS are analogous to heat-assisted magnetic recording (HAMR). The idea is to heat the free layer of the MTJ by adding current so as to lower its magnetic anisotropy and thus lower the switching current. In the case of STT-MRAM, the alignment of the electron spin towards the magnetization orientation is controlled by means of a spin-polarized current (see Figure 5c). Re-polarization of the spin can then be achieved by directing the spin-polarized current into another magnet. As a result, an STT-MTJ can be switched between a low-resistance state and a high-resistance state by using the spin-polarized current induced between the free layer and fixed layer. This means that STT-MRAM produces a relatively small switching field and consumes much less energy than conventional MTJ-based MRAM. The metal line required in conventional MRAM is also unnecessary, making STT-MRAM far more scalable.

**Figure 6:**
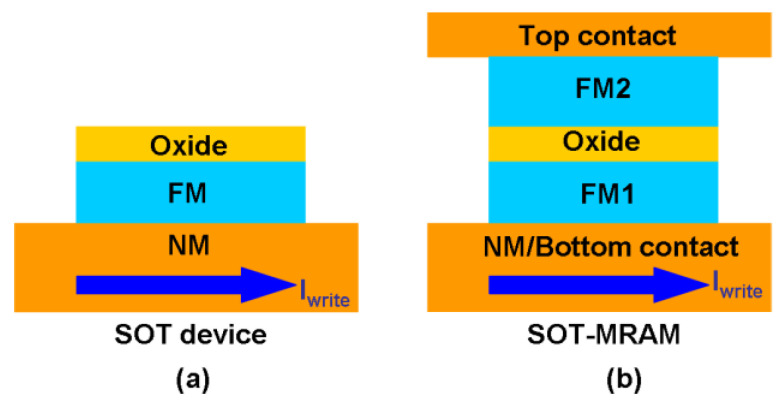
Schematic of (**a**) an SOT (‘spin-orbit torque’) device illustrating the write current path in the SOT scheme and (**b**) a SOT-MRAM cell utilizing the SOT scheme for writing and TMR (tunnel magneto-resistance) scheme for readout. FM and NM denote the ferromagnetic layer and non-magnetic layer, respectively. (Reprinted with permission from [71]).

Recently, some technical issues with STT-MRAM have been uncovered. Basically, if the write current density passing through the MTJ is too large, it can result in a breakdown of the ferromagnetic oxide. Assessing the MTJ’s magnetic state involves sensing the resistance of the MTJ stack using the tunnel-magneto-resistance (TMR) effect. However, the coupling of the read-write path in the MTJ in STT-MRAM could result in accidental switching (write) of the cell during the read operation causing ‘read-disturbance’ issues [71]. This has led to the development of an alternative approach to manipulating the magnetization, called ‘spin-orbit torque’ (SOT) [72,73,74,75,76,77]. This is able to generate a spin current that consumes less power and provides more rapid switching than an STT [78,79,80,81,82]. As shown in Figure 6, SOT devices consist of a bilayer made of a ferromagnet and a non-magnetic material, topped with an oxide. The application of an in-plane charge current to the bilayer generates a transverse spin current at the bilayer interface. This results in turn on torque being exerted on the magnetization of the ferromagnet, which can switch its magnetization. SOT-MRAM cells offer the key advantage over STT-MRAM of decoupling the read and write current paths through the MTJ, thus eliminating the risk of read disturbance. The absence of a large write current in the MTJ also adds to device stability [71]. In addition, recent research suggests that SOT-MRAM devices are easier to manufacture and can be rendered CMOS compatible [83]. Even more recently, some work has started to explore the possibility of creating STT-SOT hybrids for MRAM technology, combining the advantages of each [82]. Generally, the earlier issues with read-disturbance have largely been solved with regard to SHE (Spin Hall Effect) MRAM that decouples write and readout paths [84] and VCMA (Voltage-Controlled Magnetic Anisotropy) MRAM that uses a reverse voltage read to suppress thermal activation across the barrier [85].

### 3.2. Potential for Logic Operations

**Figure 7:**
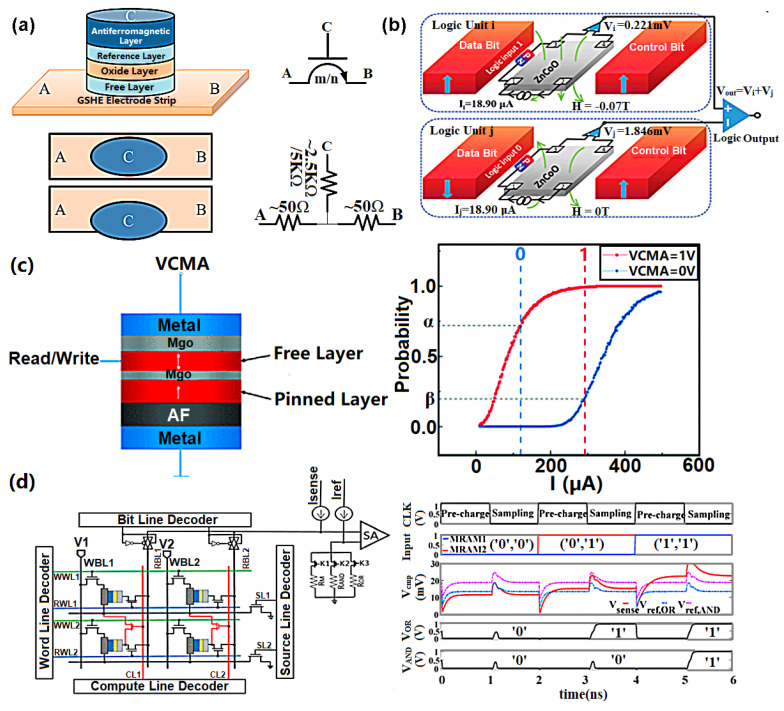
(**a**) The structure of a GSHE MTJ (Giant Spin-Hall Effect MTJ) (3D view (top left), top-down view (bottom left), device symbols (top right), and equivalence circuit (bottom right)); (**b**) schematic of the two-input logic function (NOR) in a DEMR (device diode-enhanced magnetoresistance); (**c**) device structure of the voltage-controlled MTJ (left) and its corresponding voltage and current signals to map Boolean logic ‘0’ and ‘1’ (right); (**d**) proposed SOT-MRAM based dual-mode in-memory computing architecture (left) and the resulting transient simulation of row-wise AND/OR computation. ((**a**) is reprinted with permission from [86]; (**b**) is reprinted with permission from [88]; (**c**) is reprinted with permission from [89]; (**d**) is reprinted with permission from Shaahin et al., 2018).

The scope to reversibly switch between high-resistance and low-resistance states makes MRAM especially suitable for logic function operations. In one scenario, different logic functions can be achieved by continuously changing the switching threshold of Giant Spin-Hall Effect (GSHE) STT-MRAM [86]. GSHE STT-MRAM involves far less programming time and energy than conventional STT-MRAM because it eliminates the incubation delay [87]. By controlling the connection direction between the input nodes, A and B, selecting the input nodes (n) and the switching threshold (m), the logic operation of a device can be collectively determined (see Figure 7a). The output resistance states for the upper level of any two devices controls the input current for each device, and, depending on the resistance states (R_H_ and or R_L_) of the two input devices, the current I_1_ + I_2_ can take three forms-R_H_-R_H_ (0,0), R_H_-R_L_ (0,1), and R_L_-R_L_ (1,1)- for the same bias voltage. Devices are assumed to be switched when the current exceeds a certain threshold. So, for example, a device can act as an ‘OR’ gate when the threshold is close to R_H_-R_L_ and as an ‘AND’ gate when the threshold approaches R_L_-R_L_.

Boolean logic functions can also be handled by diode-enhanced magnetoresistance (DEMR) devices [88] (see Figure 7b). The logic unit inside a DEMR device has two L10-FePt magnets with perpendicular magnetocrystalline anisotropy, which are regarded as a data bit and a control bit, respectively. Different combinations of the magnetization direction of the control bit and the data bit, i.e., ‘up-up’, ‘up-down’, ‘down-down’, and ‘down-up’, give rise to different stray fields. The magnetization direction of the data bits (i.e., ‘down’ and ‘up’) can be considered as the logic inputs ‘0’ and ‘1’, respectively. The output voltage as a sum of the measured voltages of the two logic units is then regarded as the logic output. The threshold voltage set to distinguish the output signal ‘1’ from ‘0’ is taken to be 3 mV. For the logic operation ‘AND’, the stray fields for the two logic units to give the logic input (1, 1) need to be 0 T, which gives rise to an output voltage greater than 3 mV (i.e., an output of ‘1’). The output voltages for the other input configurations are always lower than the threshold value, implying an output signal of ‘0’. By controlling the control bit and the working current, 15 Boolean logic functions can be realized.

Some recent work has explored the potential use of MTJs for stochastic computing [89,90,91]. Zhang et al. [89], for instance, have explored the use of voltage-controlled MTJs as both stochastic number generators and in-memory logic devices, with changes in voltage and current controlling the operating mode and the assignation of different Boolean logic inputs [89], as demonstrated in Figure 7c. Lv and Wang [90] have shown how the physical properties of a single MTJ, such as pulse amplitude, bias field, bias current, pulse width and switching probability, can be used for a variety of logic operations. Voltage-controlled MTJs are claimed to be more energy efficient than conventional MTJs [89] and others have indicted the scope to use switching mechanisms such as STT to further enhance the scope and energy benefits of taking this approach [90]. Stochastic computing-based logic operations offer a number of potential advantages, including not just the evident speed and energy advantages of in-memory computing but also improved accuracy and fault tolerance [91].

**Figure 8:**
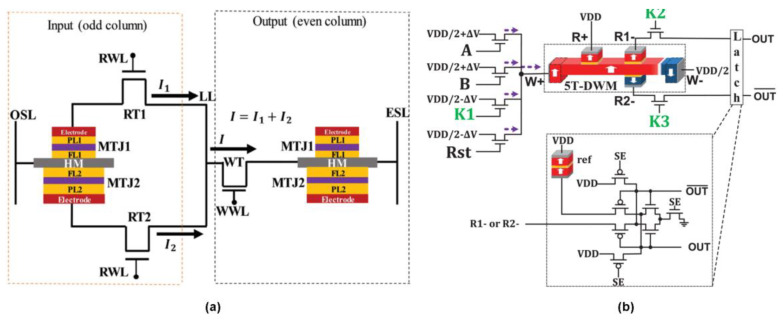
(**a**) Two input logic gate realization using MV-SOTM (SOT-based magnetic memory) in-memory computing architecture; (**b**) proposed hybrid polymorphic logic gate (HPLG) circuit design based on a 5-terminal domain wall motion device. ((**a**) is reprinted with permission from [96]; (**b**) is reprinted with permission from [97]).

Research has also begun to explore specific opportunities offered by SOT-MRAM for logic operations [89]. These build primarily upon the previously noted advantages of SOT-MRAM, namely a higher switching speed and lower energy consumption, especially the latter [92]. One specific architecture, as illustrated in Figure 7d, uses SOT-MRAM to build Boolean logic operations through the relationship between operands on the same row or in the same column within memory arrays [93]. Simulations suggest that this approach can reduce power consumption by 56% and increase speed by 31.6% in relation to other comparable NVM architectures. With regard to the potential limitations of STT-MRAM in handling big data applications, recent explorations have also begun to look at how SOT-MRAM might be used for the development of convolutional neural networks (CNNs) [94]. In related research, explorations have also been made regarding the potential use of spin current-based circuits and devices, also known as All Spin Logic (ASL) devices, as a replacement for conventional Arithmetic Logic Units (ALUs) [95]. Simulations, here, do not show the same energy savings as SOT-MRAM, but do display greater controllability. In-memory logic function has most recently been realized in a so-called multilevel voltage-controlled SOT-based magnetic memory (MV-SOTM) [96], as illustrated in Figure 8a. The in-memory logic circuit comprises two MV-SOTM memory cells that are initially employed to store the input states (i.e., memory mode). Its in-memory computing architecture is divided into odd column reserved for reading the input data from MTJ of the respective memory cell and even columns where resistance state from the free layers of the output cell is reserved and can be switched by the current passing through the respectively heavy metal. Another emerging in-memory logic MRAM arises from a hybrid spin-CMOS polymorphic logic gate (HPLG) (Figure 8b) implementing a novel 5-terminal magnetic domain wall motion device [97]. The feasibility of achieving a full set of 1- and 2-input Boolean logic functions (such as NOT, AND, and OR) based on such a device was demonstrated by configuring the applied keys [97].

### 3.3. Comparative Advantages and Disadvantages

When compared to other non-volatile memories, STT-MRAM provides the closest switching speed (<10 ns) and endurance cycles (>10^12^ cycle) to static RAM (SRAM) [57]. However, despite these encouraging properties, commercialization of STT-MRAM as a substitute for SRAM has yet to be properly realized. This is because, given the small on/off ratio (defined as the tunneling magneto-resistance ratio, or TMR ratio), the existing reading schemes need to be substantially improved. The physical performance of STT-MRAM is also strongly determined by its fabrication process, which can affect the encapsulation, etching quality and substrate smoothness [98]. Thus, advanced device and material innovations, such as Voltage-Controlled Magnetic Anisotropy (VCMA)-based MTJs [99] and heavy metal-based GSHEs [75], are urgently required to improve the magnetic and electrical properties of MTJs. A key development in this area is the move towards SOT-MRAM-based devices. This shows definite potential regarding switching speed and energy-saving. However, research in this area is nascent and all of the studies so far have generated results on the basis of simulations. There have been a few of demonstrations of the feasibility of fabricating SOT-MRAM devices on the basis of CMOS-compatible industrial processes [100], but many design considerations still need to be addressed, so it remains to be seen as to whether SOT-MRAM will live up to its promise.

The principal advances in relation to MRAM-based technology are associated with new developments around STT-MRAM and SOT-MRAM and hybrids of the two. The challenges confronting its use are that all of these new approaches are still in their infancy and relatively unproved in large-scale practical applications.

## 4. Logic in Phase-Change RAM

### 4.1. The Technology

**Figure 9:**
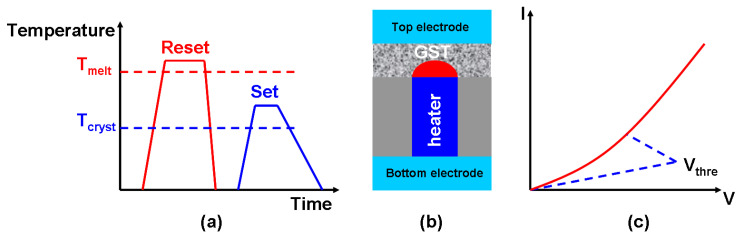
PCRAM and its characteristics: (**a**) its ‘reset’ and ‘set’ operations; (**b**) the architecture of a ‘Lance’ type PCRAM; (**c**) its typical I-V characteristics.

As a storage medium, Phase-Change RAM (PCRAM) is mainly based upon chalcogenide (Group VI) and pnictide (Group V) elements, in the form of Germanium-antimony-tellurium (GST) alloys such as Ge_2_Sb_2_Te_5_ (usually abbreviated as GST) and compositional variations of GeSb [101], GeTe [102], InSbTe [103], InGeTe [104], InSbGe, AgInSbTe [105], GeSbSeTe, GeSbReBi, SiSbTe [106], and SbTe [107]. The particularly relevant feature of these compositions, here, is that their electrical/optical properties, such as their electrical resistivity and optical reflectivity, differ sharply between their crystalline state (with a long-range atomic order) and their amorphous state (with a short-range atomic order). These two distinct states can be rapidly and reversibly switched between on the basis of temperature, making them what are known as ‘phase-change materials’ (PCMs). As shown in Figure 9a, their highly resistive amorphous state can be achieved (i.e., as a reset process) by heating their crystalline form to an appropriate melting temperature, followed by a quick cooling process. Heating amorphous PCMs to a glass transition temperature then allows for the formation of a low resistive crystalline state (i.e., as a setting process). The crystalline and amorphous states of PCMs can be defined in terms of the binary codes ‘1’ and ‘0’. The phase-transformation between their amorphous and crystalline states, which is generally induced by current pulses of an appropriate magnitude and width, is then open to being considered as the PCRAM record/write operation. The readout operation is performed by sensing variations in the resistance between phase-transformed and non-transformed regions. This is achieved by applying a readout pulse with a much lower magnitude than a write pulse.

The success of PCRAM as a non-volatile memory (NVM) technology stems from the pioneering work of Stanford R. Ovshinsky, who developed the current benchmark for PCRAM cells, known as the Lance structure [108]. It can be seen in Figure 9b that a Lance-type PCRAM consists of a GST layer sandwiched between a top electrode, usually made of metal, and a TiN resistive electrode (also called the heater). The write current pulse is applied vertically from the bottom electrode to the top electrode. It flows through the heater and the GST and the resistive Joule heating results in a ‘mushroom’ like phase-change at the heater/GST interface as soon as the temperature reaches the crystallization or amorphization point. The readout in a Lance-type PCRAM is achieved by applying a low voltage and detecting the current through the cell. The current pulse is usually produced by a cell selector such as MOSFET (Metal-Oxide-Semiconductor Field-Effect Transistor). To generate sufficiently high current density for phase transformation (particularly amorphization), it is necessary to have a fairly large cell selector. Unfortunately, this tends to diminish the integration density. A possible solution is to reduce the PCRAM programming current. This has led to the development of various PCRAM architectures that differ from the ‘Lance’ type in some way, such as the μTrench [109], ring [110], pore [111], line [112], and dash [113] architectures. These improved configurations not only significantly reduce the reset current, but also bring with them various other advantages, such as larger-scale phase transition, higher crystallization temperatures (which means longer retention), faster switching speeds, and improved endurance at smaller dimensions. 

Generally, GST has played an important role in the development of integrated all-optical PCMs [114]. Implementing ordinary integrated all-photonic PCM at a chip level has to date proved problematic because of the need to a constant bias power for the memory to be viable. However, recent work has demonstrated that, by using a GST-based non-volatile switch, on-chip all-photonic read and write operations are realizable [115]. Broadband non-volatile photonic on-chip switches have also recently been developed that can significantly surpass all existing figures of merit for performance for broadband switches, opening up the possibility of major improvements in the performance of telecommunications networks [116]. Other important trends include the development of interfacial phase change memories [117] and GSST-based photonic artificial synapses (Ge-Sb-Se-Te) [118] and the use of broadband transparent materials to enhance the performance of non-volatile photonic memories [119]. As a result of all these recent developments, many of which outperform electronic-based paradigms. PCRAM is arguably the most advanced NVM technology. 

### 4.2. Potential for Logic Operations

**Figure 10:**
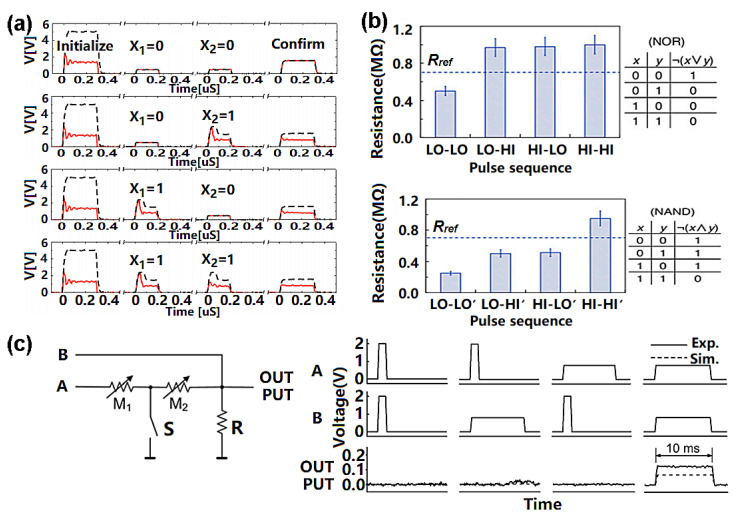
PCRAM-based logic operations: (**a**) measuring the applied voltage and voltage across the cell during initialization and computing for the four cases for logic inputs X_1_ and X_2_; (**b**) cell resistance with respect to the pulse sequence for NOR and NAND operations, respectively; (**c**) the AND setup and its corresponding voltage output. ((**a**) is reprinted with permission from [120]; (**b**) is reprinted with permission from [121]; (**c**) is reprinted with permission from [122]).

As with FeRAM, PCRAM can offer logic functions by assigning binary codes to different physical states or pulse amplitudes. One possibility is to define the PCM threshold voltage (the GST in this case) as the output signal [120]. The threshold voltage has a characteristic value and the electrical resistance of amorphous GST media undergoes an abrupt decrease if the applied voltage across the GST layer exceeds that threshold (see Figure 9c). At the same time, a pulse with a magnitude below the crystallization value can be considered to be an input signal ‘0’ and a pulse with a magnitude over that value can be treated as an input signal ‘1’. Thus, a sequence of two successive low-input pulses can leave the GST in its amorphous phase but give rise to a high threshold voltage value, representing ‘1’. The combination of one low input and one high input will result in partial crystallization and reduce the value of the threshold voltage, this corresponding to an output value of ‘0’. Two high input signals will lead to the full crystallization of the GST layer and drive the threshold voltage close to 0. This generates the logic operation ‘NOR’. Such a device can also generate a ‘NAND’ operation. This paradigm, which is shown in Figure 10a, has encouraged the development of a number of new approaches to in-memory logic operations. For instance, instead of defining the threshold voltage as a logic output, one can denote the low and high resistance of the PCRAM device as the binary codes ‘0’ and ‘1’ and assign the input logic ‘0’ and ‘1’ to write pulses with low and high magnitudes, respectively, as illustrated in Figure 10b. So, applying two consecutive input pulses with a low magnitude will leave the GST in its crystalline state, leading to a low resistance with an output value of ‘0’. Other cases that will include at least one input signal with a high magnitude will lead to the amorphization of the GST and give rise to a higher resistance. This pulse combinations can serve as ‘OR’ functions and as other logic functions such as ‘NOR’ and ‘NAND’ [121].

Note, however, that the two input signals described above work in a series rather than in parallel. The latter is often adopted in conventional CMOS circuits to increase the computing speed. To overcome this limit, a novel PCM-based logic gate supporting parallel computing was proposed in [122]. This took advantage of T-shaped cells (TiW/GST/TiW/SiO_2_/Si). As with the previous design, the ‘RESET’ and ‘SET’ pulses were used to represent the logical inputs ‘0’ and ‘1’, respectively, while the low- and high-resistance states of the PCRAM cell were used to indicate the logical outputs ‘1’ and ‘0’, respectively. The associated circuits (see Figure 10c) consist of two PCM cells and a load resistor. The node between the two cells is grounded via a switch. A write operation is performed by closing the switch and inputting the pulses at terminals A and B, while the output resistance is measured by opening the switch and applying a small signal between terminal A and the output port. Evidently, the output resistance will reach a lower state only if the two input pulses are both set to be ‘SET’ pulses. Input signals, including at least one amorphization pulse, will give rise to a higher device resistance, matching the ‘AND’ operation. Different logic functions, such as ‘OR’ and ‘NOT’, can be obtained by changing the way in which the cells, switches and load resistors are connected.

**Figure 11:**
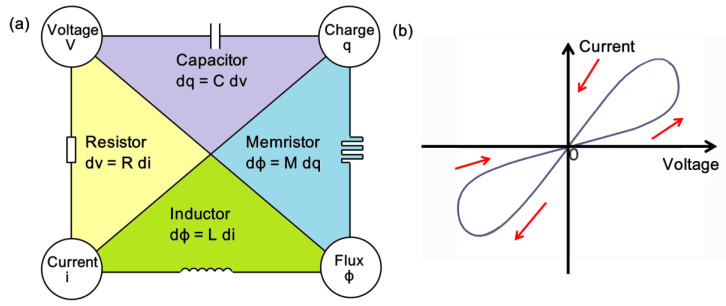
(**a**) Four fundamental two-terminal circuit elements: resistor, capacitor, inductor and memristor; (**b**) a typical pinched hysteresis loop of the memristor.

An important part of the story of the development of PCRAM is the development of what are known as ‘memristors’, literally ‘memory resistors’. These were originally theorized by Chua in 1971 [123] to be one of four fundamental electrical components, incorporating resistors, capacitors, inductors and memristors, as illustrated in Figure 11a. A memristor is effectively a two-terminal electrical component that provides for a dynamic non-linear relationship between electrical charge and magnetic flux. The characteristic of memristance associated with memristors amounts to being a charge-dependent resistance. A material that demonstrates the ideal properties of a memristor has yet to be found. However, in 2008, some 37 years after its initial conception, a nanoscale TiO_2_ device that mimicked the key characteristics of a memristor was developed [124] and a few years later the first functioning memristor arrays began to be constructed [125]. The most important feature of a memristor of interest here is its pinched hysteresis loop [126], where changes in the slope of the curve relate to different states of resistance (Figure 11b). This presents the possibility of rapidly switching between these different states as part of a two-terminal resistance memory. Basically, various hysteresis loop shapes can be acquired by changing the frequency or amplitude of different input signals. Centrally, the state variable of a memristor retains information about the electrical charge and magnetic flux, rather than just preserving the charge or flux itself. So, when the power is turned off, in theory at least, a memristor is able to remember its most recent state. This is the core reason why memristors offer the possibility of non-volatile storage of information. Through changes in input excitation, a memristor’s resistance is open to being dynamically modified, raising the prospect of memristors simulating the behavior of brain synapses.

**Figure 12:**
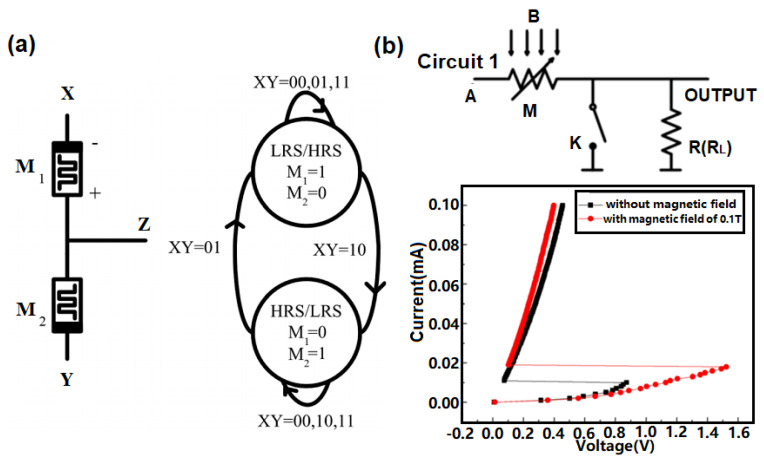
Other PCRAM-based approaches: (**a**) a memristor structure for logic operations with its corresponding state transition diagram; (**b**) an AND gate and the threshold voltage of phase-change materials with and without a magnetic field ((**a**) is reprinted with permission from [127]; (**b**) is reprinted with permission from Lu et al., 2016).

This idea was further expanded to a circuit containing two anti-serially connected memristors with a Ta/GeTe/Ag structure [127] (see Figure 12a). The logic operations, here, depend on the fact that the device resistance increases when the current flows from negative to positive polarity, while a flow from positive to negative will decrease the resistance. For logic operations, two memristors can be connected back to back and the logic variables A and B can be applied to the terminals X and Y, respectively. By using this configuration, a positive SET voltage will result in a highly resistive state, with a low resistive state in the top and bottom memristors. A negative RESET voltage, by contrast, will switch the top and bottom memristors to a low and high resistive state, respectively. This implies that setting and resetting the two memristors will allow for the storage of the logic values ‘0/1’ and ‘1/0’, respectively, thus enabling the realization of all Boolean logic operations.

It should be noted that although memristors were originally developed in the context of PCRAM, they have also played (and continue to play) an important role in relation to both FeRAM and STT-MRAM. Although they are distinct, there is also an affinity between the idea of a memristor and ReRAM, so they are strongly related [128].

Recently another PCRAM structure that uses a [GeTe/Sb_2_Te_3_]_n_ super lattice PCM has been proposed that is also capable of in-memory logic operations [129]. The proposal arose from the finding that applying an extra magnetic field to a super lattice PCM can modify its threshold voltage. The resulting circuit has two input terminals, one for the voltage pulse and one for the magnetic field (see Figure 12b). A voltage pulse with a magnitude of 3 V/0.5 V corresponds to the logic value of ‘1’/’0’ for one input terminal, whereas the presence/absence of the magnetic field represents the logic value ‘1’/’0’ for the other input terminal. If no magnetic field is applied, neither of the input signals will electrically switch the super lattice PCM, giving rise to a high device resistance (i.e., an output logic value of ‘0’). The device resistance undergoes a drastic reduction only if the input pulse is increased to 3 V, together with an applied magnetic field. This triggers the ‘AND’ gate and other logic gates such as NAND’, ‘OR’, and ‘NOR’. Most recently a complete in-memory hyperdimensional computing (HDC) system was proposed to accomplish a near learning optimum trade-off between design complexity and classification accuracy [130]. Such in-memory HDC mainly consists of an item memory (IM) that stores *h*, *d*-dimensional basis hypervectors and an associative memory (AM) that stores *c*, *d*-dimensional prototype hypervectors. One encoder was implemented to perform dimensionality preserving mathematical manipulations during learning and to generate a query hypervector during classification. Both IM and AM inside the in-memory HDC are arranged in the form of crossbar arrays of memristive devices to achieve the comparable accuracies to software implementations. 

**Figure 13:**
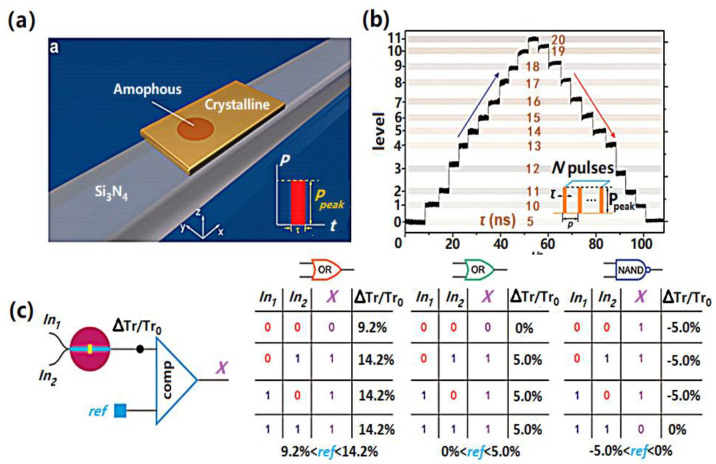
Photonic-based PCRAM: (**a**) an all-photonic memory structure; (**b**) the resulting transmission levels when adjusting the intensity and the width of the optical pulse; (**c**) schematic of the optical logic device and its corresponding ‘truth’ table. (Reprinted with permission from [131]).

Apart from the above, phase-change memories can also be used optically to realize Boolean logic functions [131] (see Figure 13). This novel all-photonic device uses GST media as the active layer, coated with an ITO (Indium Tin Oxide) film to protect it from oxidation. These two layers are deposited over a half-etched waveguide on a Si_3_N_4_ substrate that is optimized for single mode operations. To record data, an intense light is propagated through the waveguide and the resulting energy, particularly the evanescent electric field, is partly absorbed by the GST media. The required phase transition can therefore be accomplished once the temperature inside the GST media reaches either ~400 °C for fast crystallization or melting point for amorphization. The readout mechanism for this photonic device relies on the significant difference in the refractive index between the crystalline and amorphous phases. Experimental results show that amorphous GST has a lower attenuation and higher light transmission than its crystalline phase. Thus, the device transmission coefficient, measured by a low power optical pulse, is significantly enhanced when forming the amorphous element, whereas a lower transmission coefficient is detected when producing a crystalline one. If this mechanism is adopted, a series of device transmission values can be obtained by continuously changing the magnitude and width of the optical excitation, enabling multi-level recording. Optical pulses with three different widths can be set as ‘reset’, ‘input 0’, and ‘input 1’. To perform logic operations, the device can initially be reset to a pre-defined transmission value using a reset signal. This pre-defined transmission value will remain unchanged unless the two input signals include at least one input value of ‘1’. ‘OR’ logic can be thus be realized by using a reference transmission between the pre-defined value and the one resulting from an input ‘1’ signal. 

### 4.3. Comparative Advantages and Disadvantages

PCRAM is considered to be the most competitive rival of Flash or even DRAM because of its scalability (<5 nm), fast switching speed (~ns), and excellent endurance (>10^11^). However, PCRAM will not replace Flash as a working memory in the shorter term because of the outstanding recent progress in Flash devices, particularly with regard to their write speed (~ns) and endurance (>10^12^). The relatively high cost of PCRAM is another factor that is hampering its commercialization. Nonetheless, when compared to its electronic counterpart, the capacity of phase-change photonic memory to take advantage of light to convey, record, and detect information, enables it to not only circumvent the bandwidth limitations of silicon electronics, but to also obviate the need for extra devices for electronic-to-light conversion. This being said, it is currently still difficult for it to provide a density comparable to conventional phase-change memories, despite the possibility of multi-level recording.

**Figure 14:**
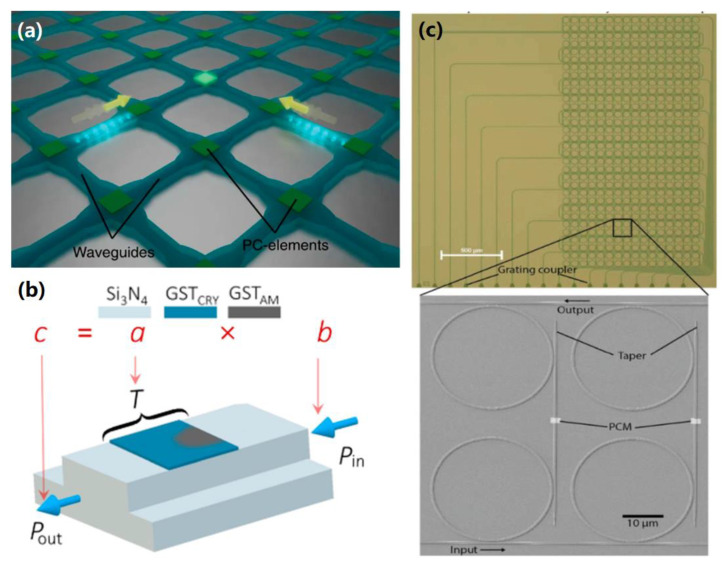
(**a**) Sketch of a waveguide crossing array illustrating the two-pulse addressing of individual phase-change cells. Only overlapping pulses provide sufficient power to switch a desired PCM-cell; (**b**) scheme of the multiplication of two scalars a and b, codified in the device transmittance T and in the energy of the read pulse Pin; (**c**) 16 × 16 cell photonic matrix memory showing optical micrograph of the memory cells (top) and SEM of a single cell within the array (bottom). ((**a**) is reprinted with permission from [133]; (**b**) is reprinted with permission from [134], and (**c**) is reprinted with permission from [135]).

Most PCM-based computational memories function electronically and use the contrast in electrical resistivity between the PCM’s amorphous and crystalline states [132]. The viability of photonic computational memory depends upon the effectiveness of the computations inside the device itself. A potential scheme (see Figure 14a) for performing arithmetic calculation is to use a rectangular waveguide array where a PCM-cell is deposited at every waveguide crossing point [133]. This makes it possible to selectively address and manipulate each basic arithmetic unit. So, as an example, base-10 arithmetic calculations can be performed by using a group of identical picosecond (ps) pulses to divide the degree of crystallization in each PCM cell into 10 different levels. The PCM can then be re-amorphized by using another group of picojoule pulses. This strategy was recently used to realize logic functions within the same photonic device while adopting a novel pulse width modulation (PWM) scheme [131]. Usually, the pulses used to induce phase-transformation adhere to a pulse amplitude modulation (PAM) scheme, where there is a fixed width and different amplitudes. PWM, by contrast, uses optical pulses with a fixed peak amplitude and different widths. Thus, the phase-transformation levels can be controlled by using different pulse widths. The key advantage of using PWM is that it makes it possible to directly access one specific memory level (i.e., phase-transformation level) from any other memory level using the same pulse sequence. This significantly increases the range of logic functions that can be realized within the PCM-based memory. This approach led to development of a photonic computational memory that can handle direct scalar multiplications of two numbers [134], using a single integrated PCM-based photonic cell (see Figure 14b). In this device, a write pulse, Pwrite, programmed the device to a particular level of transmittance (T). Another, lower intensity, read pulse, Pin, was then used to sense the device transmittance without changing the PCM’s phase-transformation. The power of the Pin pulse at the output port, Pout = T(P_write_) × P_in_, is the result of an a × b multiplication, where the multiplicand a is mapped to T and the multiplier b is mapped to P_in_. 429 multiplications using arbitrary values were undertaken using this method and matched with the exact multiplication values. This underscores the potential of PCMs for developing photonic hardware. Critically, it demonstrates the real scope to integrate optics, data storage and processing in a single all-photonic memory that is capable of in-memory computations.

A key problem confronting the development of all-photonic memory circuits is making them scalable. Recently, however, Feldmann et al. have developed a 256 cell all-photonic phase-change memory that can store 512 bits of data [135], with the data being stored in an array of nanoscale PCM-based devices. This memory array is based upon rows of microring resonators that are individually connected to a single input waveguide (see Figure 14c). Every row has a certain number of memory cells consisting of an input and output waveguide, joined by two microring resonators, with another waveguide with a PCM-patch on top. As light passes through the input waveguide, part of it is coupled to the lower microring resonator and PCM waveguide. Once it has passed through the PCM and induced phase-transition, it is guided to the second microring resonator. The input wavelength can be adjusted by tuning the radius of the microring resonators. As both resonators have the same radius and resonance wavelength, the input signal can easily be multiplexed and demultiplexed. The quantity of light going to the PCM and output waveguide can be controlled by adjusting the gaps between the resonators and the waveguides. The actual data storage component is the PCM cell between the resonators. This design was tested using SiN-based waveguides and resonators, a GST PCM, and a protective ITO layer and was able to store a 16 × 16 pixel pictogram of a floppy disk in two-bit resolution. All of the cells could be addressed individually and could reproduce the pictogram with a high degree of accuracy. This device had an overall footprint of 1000 × 2400 μm^2^.

Conventional optical computing approaches have been limited by the lack of an integrated non-volatile photonic memory and multiplexing capability for calculations. Recent work has shown how this can be alleviated by using an integrated photonic tensor core that consists of phase-change memory arrays that can locally store convolution kernels on-chip [136], together with photonic chip-based frequency combs that can provide in-memory photonic computing using wavelength division multiplexing (WDM). Calculations are performed by measuring the reconfigurable and non-resonant optical transmissions. This photonic device has the potential to conduct computations at the speed of light while consuming only tiny amounts of power. This method promises to eliminate the existing computing bottleneck in machine learning hardware and could be used for applications ranging from live video processing to autonomous driving and AI-assisted life-saving. In other recent work, Prucnal et al. have developed a silicon-based photonic network (consisting of digital electronics and analogue photonics (DEAP)) that can be used for convolutional neural networks [137]. The network is capable of performing convolutions at between 2.8 and 14 times faster than a GPU while using approximately 25% less energy. To test its viability, it performed a convolution and solved an MNIST handwriting recognition task with an overall accuracy of 97.6%. Importantly silicon photonics-based computing memory has the capacity to both outperform the conventional electronic hardware used for machine learning and to be potentially scalable for a range of future applications.

Further recent developments in PCM-based computational RAM at IBM have demonstrated the potential of PCRAM to store synaptic weights, taking things a step closer to brain-like memory and processing [132,138]. This technology has been shown to be able to handle cloud-based two-layer neural network processing of relatively large bodies of data [132] and seems to also have the potential to handle more complex, convolutional neural networks [139].

The challenges confronting the ongoing development of PCRAM and its uptake are, as with MRAM, the relative immaturity of many of the most promising technological approaches and, closely related to this, the currently high cost of the materials required for its implementation.

## 5. Logic in Resistive RAM

### 5.1. The Technology

**Figure 15:**
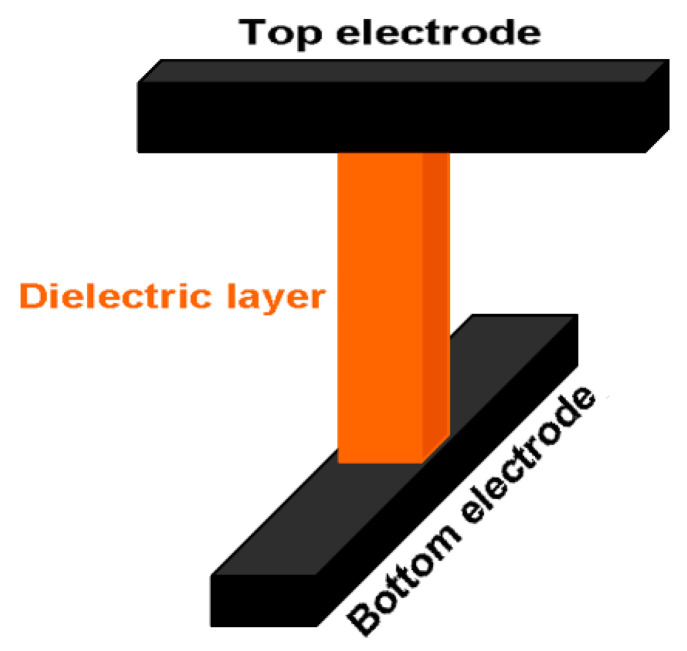
Typical RRAM architecture.

**Figure 16:**
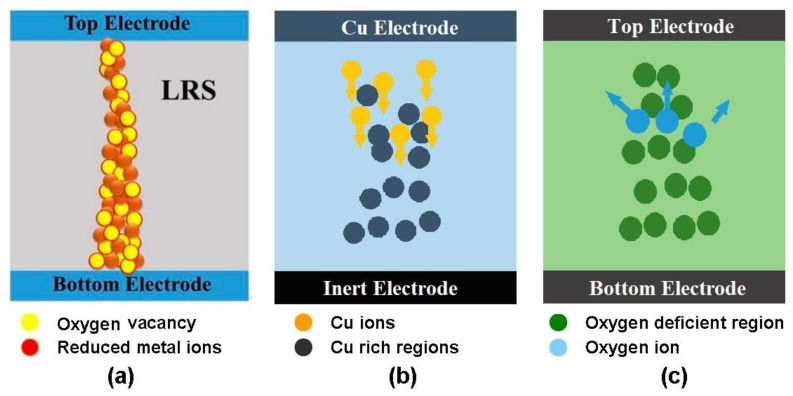
Resistive switching mechanism for: (**a**) VCM (valence change memory); (**b**) ECMM (electrochemical metallization memory); and (**c**) thermochemical memory ((**a**) is reprinted with permission from [148]; (**b**,**c**) are reprinted with permission from [64]).

Resistive RAM (RRAM or ReRAM) basically makes use of a simple metal-insulator-metal (MIM) architecture (see Figure 15), with a dielectric layer being sandwiched between two metal electrodes. The most attractive feature of RRAM is that a dielectric layer that is subjected to external electrical excitations can be reversibly and rapidly switched between high and low resistance states, just as is the case with PCRAM. There is still some controversy surrounding the resistance switching (RS) phenomena found in various resistive materials, such as transition metal oxide, organic polymer composites, graphene oxide, and selenides. Several hypotheses have been put forward regarding the RS mechanism of these materials, including valence change memory (VCM) [140,141,142], electrochemical metallization memory (ECMM) [143,144,145], and thermochemical memory [146,147,148] (see Figure 16). 

Devices that are assumed to work on a VCM basis mainly adopt oxide-based materials to generate a large amount of oxygen vacancy when subjected to an external electric field. This causes a change in the valence of the cations in the active RS layer. The migration of the oxygen vacancies and metal cations leads to the formation of a conductive filament through the entire RS layer, allowing for LRS (Low Resistive State). To restore an HRS (High Resistive State), higher levels of electrical excitation are employed to rupture the conductive filament, forcing the metal ions to be oxidized and the number of oxygen vacancies to decrease.

ECMM cells have a solid electrolyte layer sandwiched between an electrochemically active electrode, such as Ag or Cu, and an electrochemically inert electrode, such as Pt or W. Unlike VCM, the formation and rupture of the conductive filament inside an ECMM is focused upon changing the polarity of the externally applied bias. Application of a positive voltage gives rise to an anodic dissolution of the metal and the resulting metal cations thus drift toward the inert electrode via the solid electrolyte. The deposition of the metal cations on the surface of the inert cathode reduces the number of metal positive ions, thereby switching the cell to an LRS. Reversing the polarity of the applied bias facilitates electrochemical dissolution of the conductive filament, switching the cell back to an HRS. As with VCM, the thermochemical memory also encourages the formation of a conductive filament for the migration of oxygen vacancies. In a thermochemical memory device, the application of a negative bias pushes the O^2−^ ions away from the top electrode, but attracts oxygen vacancies towards it, thereby forming a filament-like path. At present, the switching process from an LRS to an HRS for thermochemical memory is considered to be the consequence of the thermal rupture of the filaments because of the heat produced in the presence of a large current flow.

RRAM has recently received a lot of attention, especially with regard to device fabrication and the kind of materials to use for the electrodes. A scalability of cells below 10 nm and a switching speed of ~100 ps have been accomplished using HfO_x_/WO_x_-based RRAM [149] and TaO_x_/Pt dispersed SiO_2_-based RRAM [150], respectively. RRAM architectures using TiN/Hf/HfO_2_/TiN and Pt/Ta_2_O_5−x_/TaO_2−x_/Pt also exhibit an ultra-low energy consumption of <0.1 pJ [151] and an extremely long endurance of >10^12^ switching cycles [152], respectively. The possibility of developing RRAM with a data retention time of several months or even up to 10 years has also been demonstrated experimentally and in simulations. These achievements indicate the scalability of RRAM, its excellent stability, and its scope for handling fast switching speeds. 

Despite these achievements, there have been concerns expressed that the matrix-vector multiplication characteristic of RRAM’s crossbar structure does not have the flexibility to meet changes in the market, especially towards big-data applications [153]. To offset this, some research has focused on developing fully programmable in-memory computing architectures. In this way, the RRAM crossbar structure can provide flexible partitioning according to the specific needs of different applications [153].

### 5.2. Potential for Logic Operations

**Figure 17:**
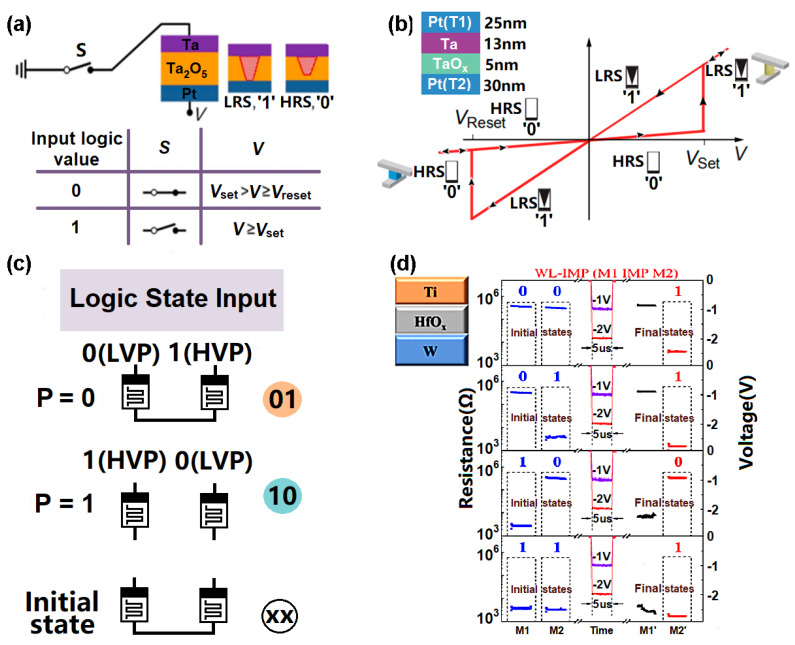
Various RRAM configurations: (**a**) a hybrid device structure with one switch and one unipolar RRAM cell; (**b**) the I-V characteristics of a typical TaOx-based RRAM; (**c**) the logic operation of two anti-serial RRAM devices; (**d**) experimental results for “WL-IMP” operations on M1 and M2 ((**a**) is reprinted with permission from [154]; (**b**) is reprinted with permission from [155]; (**c**) is reprinted with permission from [156]; (**d**) is reprinted with permission from [157]).

In relation to in-memory logic operations, the LRS and HRS of RRAM perfectly match the binary digits ‘1’ and ‘0’, renders it suitable for in-memory computation. One practical scheme to realize in-memory computing of logic operations for RRAM is to develop a hybrid device with one switch and one unipolar Ta_2_O_5_-based RRAM cell [154] (see Figure 17a). The switch’s physical state and the external voltage can be considered as input logic signals. Thus, the ‘on’ and ‘off’ states of the switch can correspond to the input logic values of ‘0’ and ‘1’, respectively, while, for the external voltage, the input logic values of ‘0’ and ‘1’ can refer to writing the RRAM cell into comparable states of ‘0’ and ‘1’, respectively. The non-volatile HRS and LRS of an RRAM cell can indicate output values of ‘0’ and ‘1’, respectively. Thus, a logic combination of input (01) can enable the switching of the RRAM from a ‘0’ to a ‘1’ state, while maintaining a ‘0’ state the other three combinations (00, 10, and 11) are encountered. Switching from an output state of ‘0’ to ‘1’ can be achieved by using the 00 input combination, while the other three input configurations will not change the memory state. This allows for the production of 14 of the 16 Boolean logic functions in no more than three sequential write cycles.

Recently, another type of RRAM was developed that makes use of a complementary resistive switch (CRS). This, too, can realize a range of Boolean logic functions [155] (see Figure 17b). The CRS circuit can be thought of as two anti-serially connected bipolar switching devices, at each cross-point junction. Each junction element is thus regarded as a two-terminal device (terminals T_1_ and T_2_), when the input logic signals are applied. Application of a high potential and a ground potential will represent logic values of ‘1’ and ‘0’, respectively. Only a certain input combination can switch the junction element, depending on the previous device state. A destructive spike readout scheme can be applied to establish the output logic signal, with the presence or absence of a spike meaning ‘0’ or ‘1’, respectively. This CRS-logic concept has been effectively demonstrated for VCM-based RRAM, using TaOx as the active material. Ta_2_O_5_-based RRAM and HfO_2_-based RRAM both have bipolar resistive switching (BRS) and CRS-related structures and are capable of realizing in-memory computation of logic operations [156] (see Figure 17c). For the BRS circuit shown in Figure 17c, a high voltage pulse and an LRS are defined as being a logical 1, while a low voltage pulse and an HRS are defined as a logical 0. Devices with an initial HRS (‘0’) state can only be switched by applying a high voltage pulse (‘1’) to the word line, with the bit line grounded (‘0’). This means that the other three combinations (00, 01, 11) cannot induce a state transition. However, switching to the logic value of ‘0’ from ‘1’ can only be obtained from the input 01, while 00, 10, and 11 will preserve the memory state. A CRS circuit can be viewed as two anti-serially connected BRS, with the logic function being realized by floating the terminal bit line and applying operation signals to the two selected word lines. Although the definition of the terminal voltage is the same as it is for the BRS, the CRS structure includes four memory states rather than the two available in the BRS. The output states 00 and 11 can be converted to 01 or 10, given the inputs 01 or 10, and the output states 01 and 10 can be transformed into each other if the right signals are applied.

**Figure 18:**
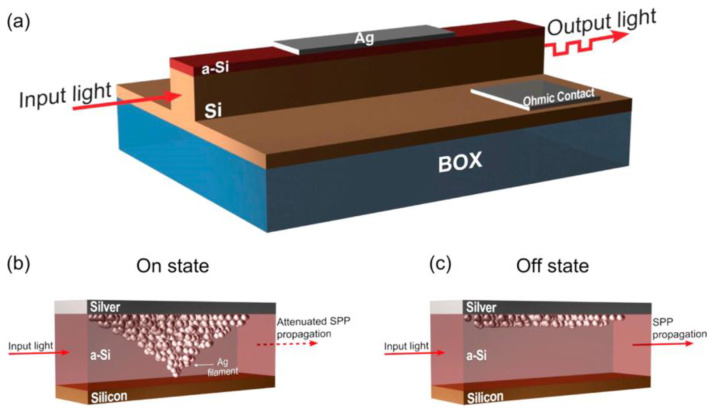
(**a**) Three-dimensional schematic of the investigated optically readable plasmonic RRAM; (**b**) formation of the nanoscale filament in the on state; (**c**) annihilation of the nanoscale filament in the off state (Reprinted with permission from [158]).

The key feature of RRAM is the similarity of its electrical behavior to that of a ‘memristor’. This endows RRAM with the capacity to realize material implication (IMP) logic. This offers a number of advantages, such as non-volatility, functional completeness, high compactness, and the symmetry of the two inputs for a binary function. Basically, two RRAM (considered as memristors here) are selected by a common shared word line and two bit lines. Applying a positive voltage higher than the ‘SET’ value and a negative voltage higher than the ‘RESET’ value can switch the RRAM from an HRS to an LRS and vice versa. The initial resistive states of the two memristors indicate the logic inputs for binary computation. During logic operations, V_COND_ and V_SET_ are simultaneously applied on bit line 1 and bit line 2, respectively. The logic result stored inside the RRAM then corresponds to its final resistance. As this scheme uses two RRAM in the same word line, it is usually called WL-IMP. A different approach to WL-IMP was recently proposed that implements two RRAM in the same bit line, leading to it being called BL-IMP. BL-IMP presents an analogue circuit configuration to WL-IMP, apart from the voltage polarity. The feasibility of using these two schemes to achieve important Boolean functions has been proven experimentally. In one notable case, a 2 × 2 Ti/HfO_2_/W RRAM array [157] was used to execute logic computation and apply logic inputs (i.e., V_COND_ and V_SET_ in either positive or negative polarity) to two working RRAM, followed by a read process to detect the correct logic output (see Figure 17d). This method enables the 16 binary Boolean logic functions to be reprogrammed in one small single cell with superb performance. Differing from the aforementioned electrically writable and readable RRAMs, one novel nanoscale plasmonic memristor that can be written and re-written electrically but read optically was also devised, as illustrated in Figure 18. The designed device comprises a vertical Ag/a-Si/p-Si butt-coupled to a single-mode SOI waveguide where light is propagated. As light propagates through such a device, it couples mainly to the fundamental plasmonic mode, highly confined at the Ag/a-Si interface. The formation and rupture of the conductive Ag filament inside a-Si can greatly change the optical transmission of the RRAM device, enabling its readout functionality [158].

Some recent research has taken a somewhat orthogonal view to other approaches by exploring the provision of a non-volatile memory architecture for logic operations that is able to work with different kinds of NVM, including phase changing memory (PCM), STT-MRAM and RRAM. One such proposition [159] has looked at redesigning the read circuitry to enable the computation of bitwise logic in multiple memory rows. This seems to offer very efficient processing-in-memory (PIM) and significantly better processing speeds and energy consumption than conventional processors [159]. In principal, memristors can be used for both logic and memory functions, thus supporting the development of resistive computing architectures. Borghetti et al. [160] demonstrated that these kinds of devices have the scope to be used for fundamental Boolean logic operations, with them being able to work simultaneously as logic gates and memory latches based solely upon resistance, rather than voltage or charge. Vourkas et al. [161] subsequently developed a new logic circuit design paradigm based upon these characteristics of memristors. Kvatinsky et al. [162] took further advantage of this idea to construct a memristor-based logic family that they called MAGIC (Memristor Aided LoGIC). They had previously used the same idea to construct a family of hybrid CMOS-memristive logic devices, so that the approach was compatible with existing CMOS logic [163]. In addition to the aforementioned achievements, a novel RRAM-based very long instruction word (VLIM) architecture for an in-memory computing (ReVAMP) system was also proposed [164]. Its data storage and computation memory (DCM) that performs data and in-memory computation makes use of RRAM crossbar memory, while the instruction memory (IM) stores the instruction and is accessed using the program counter (PC). The proposed work outperforms state-of-the-art programmable logic in-memory (PLiM) architecture by up to 11.2× in terms of latency. A robust in-memory computing core with digital input and analog output multiplication-and-accumulation (MAC) circuit based on RRAM was also designed, leading to 2.23−7.26× better energy efficiency in an 8-bit weight pattern when compared with previously reported in-memory computing implementations and deep learning accelerators.

**Figure 19:**
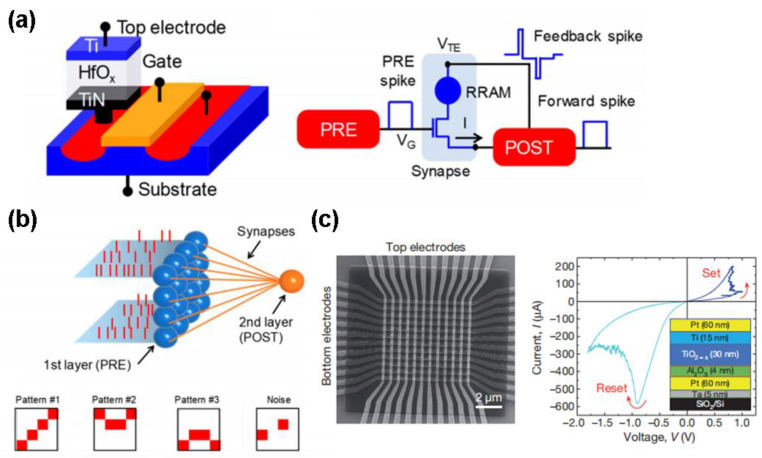
(**a**) Schematic structure of the 1T1R RRAM structure (left) and of the 1T1R as a synapse to achieve STDP (spike-timing-dependent-plasticity) in hardware (right); (**b**) schematic of a single-layer perceptron network where a 4 × 4 input layer is fully connected to a single post-synapse (top) and sequence of three visual patterns submitted to the neural network during training process and an example of a random noise image, which is alternatively applied to patterns according to a stochastic approach (bottom); (**c**) integrated 12 × 12 crossbar with an Al_2_O_3_/TiO_2-x_ memristor at each crosspoint (left) and a typical current-voltage curve of a formed memristor (right). ((**a**) is reprinted with permission from [165]; (**b**) is reprinted with permission from [166]; (**c**) is reprinted with permission from [168]).

Another fascinating feature of RRAM arises from its ability to construct the so-called spike neural networks (SNNs) that can closely imitate the storing and computing functionalities of the biological brain. One strategy is to take advantage of a Ti/HfOx/TiN RRAM based on 1T1R configuration [165], as illustrated in Figure 19a. The possibility of realizing the well-known spike-timing-dependent-plasticity (STDP) behavior using the designed 1T1R synapse was also verified. In addition to the emulation of the STDP mechanism, the RRAM-based SNNs were also implemented for unsupervised learning of visual patterns in hardware [166], as shown in Figure 19b. Pattern learning experiment is based on three sequential phase where only one 4 × 4 visual pattern among Pattern #1, Pattern #2, and Pattern #3 is submitted to the input layer, and was conducted using a stochastic approach. Note that above RRAM-based SNNs generally adopt a concept of overlap-based synapse. Recently, a concept of non-overlap ReRAM-based synapse was also presented [167], exemplified by a Pt/Ta_2_O_5-x_/TaOy/Pd RRAM device. Adjusting the sequence of the pre-and post-synaptic spikes gives rise to the analogous STDP behavior to the observed biological phenomenon as well as the numerically calculation outcomes. In spite of these impressive studies, hardware implementation of the neuromorphic system still remains challenging due to the extremely complexity of the human brain that contains ~ 10^14^ synapses. One promising scenario to provide the similar degree of complexity is to implement a so-called cross-bar architecture where a two-terminal resistive device (i.e., memristor) is located at each cross-point. It should be noticed that conventional cross-bar neural networks still adopt an additional transistor at each cross-point, which severely impairs their scalability. To overcome this limit, a transistor-free metal-oxide memristor with Pt/Al_2_O_3_/TiO_2-x_/Pt stack [168], as described in Figure 19c, was recently reported to achieve a successful operation of a simple integrated neural network having one single layer perceptron, thus paving the path toward the effective analog-hardware realization of much more complex neuromorphic networks. 

### 5.3. Comparative Advantages and Disadvantages

Although RRAM offers a fast speed, long endurance, and large on/off ratio, its retention time remains a problem. This is largely a result of RRAM devices exhibiting a rapid ‘relaxation’ behavior immediately after programming, thus undermining their reliability and retention capacity [169]. The switching mechanisms for RRAM also rely on the formation and rupture of a localized conductive filament through insulating dielectrics. The location, dimension and composition of the filaments differ from cycle to cycle and from cell to cell. This causes an intrinsically stochastic switching process that leads to fluctuations in the device resistance and switching voltage [170]. Most critically, the majority of RRAM devices require a forming process (i.e., placing under a higher bias or for a longer duration than the set/reset conditions) prior to them being able to realize stable programming. This leads to added complications in the device design and operation [163]. How to construct a truly ‘forming-free’ RRAM is still unknown. Thus, the principal challenges confronting RRAM relate to the need to produce more stable and durable solutions with a much lower manufacturing overhead.

## 6. Conclusions

A completely brain-like device needs to be able to simultaneously process and store data in the same place. This is beyond the capability of conventional CMOS-based circuits. The advent of non-volatile devices has presented the possibility of realizing genuinely neuromorphic computers. This is mainly down to their resistive switching characteristics, which enable computation and storage to happen not only in the same place, but also at the same time. The physical attributes of these devices, such as their scalability, fast switching speed and low energy consumption, make their computation and storage behavior very close to that of biological synapses. However, to seriously compete with existing established forms of volatile memory, non-volatile memory has a number of issues to overcome. Comparing the relative advantages and disadvantages of the various approaches to non-volatile RAM we have discussed above and their scope to replace volatile memory, we can note the following:

- FeRAM offers low power consumption, a fast writing speed and good endurance but it lacks scalability. The latter is largely due to the limitations of the ferroelectric materials currently being used and this has become the main focus of research in this area, with some materials showing definite promise, such as metal oxide-doped HfOx [56,57]. A distinctive approach to tackling the scalability issue is to replace capacitor-type FeRAM with Fe field-effect transistors (FeFETs) [58,59,60], though this can impact rewrite capacity and retention time, necessitating the use of yet other materials. Ferroelectric tunnel junction (FTJ)-based devices also show promise in this area [10,63], though early tests of their potential to deliver large on-off tunnel current ratios [54] require further confirmation.
- Some forms of MRAM, for instance STT-MRAM, offer performance that is comparable, at least in terms of switching speed and endurance, to more mainstream forms of volatile RAM, such as SRAM [56]. However, other features of their performance such as their TMR ratio and reading schemes are preventing them from being properly commercialized. Additional fabrication issues indicate a need for more advanced designs and materials for MRAM, for instance, Voltage-Controlled Magnetic Anisotropy (VCMA)-based MTJs [97] and heavy metal-based GSHEs [98]. There have been notable successes in the fabrication of SOT-MRAM-based devices that overcome many of the issues associated with STT-MRAM [99], but it is clear that current density and field-free switching are still issues that will require further work and most research in this area remains largely theoretical at present.
- PCRAM offers the closest non-volatile memory performance to volatile memories such as Flash and DRAM, in terms of not only switching speed and endurance but also scalability. However, high manufacturing costs make it unlikely that PCRAM will be treated as a serious competitor to Flash in the shorter term. If it can overcome its present density limitations, perhaps the most promising development in PCRAM is phase-change photonic memory, because it can use light to convey, record, and detect information, thereby circumventing the bandwidth limitations of traditional electronics and stepping around any need for extra devices for electronic-to-light conversion.
- RRAM performs well in terms of its speed, endurance and TMR ratio, but has issues in terms of retention capacity and reliability [169]. RRAM also makes use of an intrinsically stochastic switching process that can cause fluctuations in device resistance and switching voltage [170]. This, coupled with the complexity arising from its need for a forming process [171] makes RRAM an unlikely replacement for conventional volatile memory in the near future. It should be noted that MRAM also has a stochastic nature. Here, however, this has formed the basis of non-volatile stochastic computing schemes, which have traded upon its stochastic nature to gain energy advantages, improve tolerance to errors and reduce complexity and cost [89]. Attempts have also been made to turn the stochastic character of RRAM to good effect in the context of stochastic computing by producing random bit streams and reshuffle bit streams at a much lower cost. These can then be used as a basis for certain kinds of solvers and clustering algorithms [172].
- When it comes to implementing the above as memory arrays, such arrays are currently focused on crossbar array architectures that typically make use of either phase-change materials (see [173,174]) or resistive materials (see [43], [175,176,177]). More work is still needed on realizing memory arrays using either FeRAM or MRAM, though there have already been some successes in constructing STT-MRAM memory arrays [178], and there have also been some notable moves in this direction using SOT-MRAM lately [179]. By and large, this paper has focused on discussing single device implementations and how they might be used for logic operations, but constructing larger-scale non-volatile memory arrays has also been given substantial treatment in the literature and significant progress has been made [180,181].

A comparison of the physical performance of non-volatile memories is shown in Table 1. It can be seen that RRAM currently exhibits the smallest feature size and shares the smallest cell area with PCRAM. Only FeRAM does not offer multilevel switching. The write-erase time for RRAM is also superior to the other devices, while PCRAM performs the worst in this respect. The retention time for all devices is much the same, while MRAM offers the best overall endurance. Examination of the table reveals that FeRAM is outperformed by at least one other kind of non-volatile memory in some respect. RRAM, by contrast, has the greatest overall superiority. 

**Figure 20:**
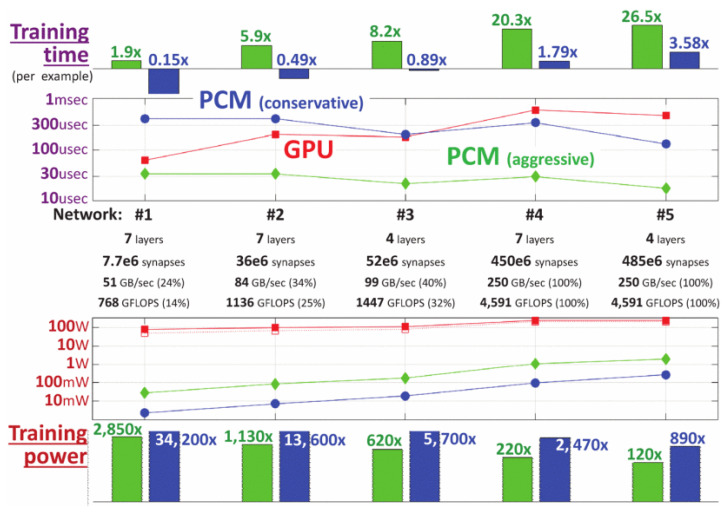
Training time and power comparisons between PCM-based on-chip machine learning and conventional GPU (Graphical Processing Unit) training. (Reprinted with permission from [186]).

It is interesting to compare the performances of the large-scale artificial neural networks (ANNs) using NVM-based synapses with those trained using CPUs or GPUs. A 3-layer perceptron with 164,885 PCM-based synapses has been developed and trained with backpropagation to recognize the handwritten digits, providing a similar test accuracy to the case when trained by software [186]. However, both training and test accuracies in these initial experiments were limited to 82–83% due to the nonlinearity and asymmetry in PCM conductance response. Such problems might be overcome by developing more advanced algorithm to offer wider tolerance to learning rate, higher classification accuracies, and lower training energy. The expected training time (per ANN example) and training power comparison between PCM-based on-chip machine learning and conventional GPU training is schematically shown in Figure 20. The PCM-based on-chip machine learning seems to enable lower power and faster training for both large and small networks than the conventional GPU training. However, either increasing the circuit sharing or performing occasional RESET too frequently may harm the speed of the PCM-based on-chip machine learning. Looking on to which device is the most promising in terms of logic operations, it should be noted that, in order to replace the traditional arithmetic/logic unit of a CPU, non-volatile memory will need to not only perform logic operations, but to also process fundamental arithmetic/matrix computations. This requires a gradual change in resistance, rather than abrupt switching. This is probably beyond FeRAM and MRAM. For this reason, too, RRAM-based devices are seen as the most promising candidates for future memory and logic computing applications. However, a lot of work still needs to be done to resolve their current issues regarding reliability, control of the switching process, variability and the complexity of their manufacture. These all urgently need further research before there will be any realistic prospect of the commercial development of RRAM devices.

## Figures and Tables

**Table 1:** Performance comparisons of different nonvolatile devices.

	MRAM	PCRAM	FeRAM	RRAM	GPP * with On-Chip Memory
Non-Volatility	Yes	Yes	Yes	Yes	No
CMOS Technology (nm)	130	45	180	< 5	65
Write/Read Time (ns)	10/10	50/10	30/10	5/5	2/2
Cyclability	>10^14^	10^6^ ~ 10^9^	10^14^	10^6^ ~ 10^9^	>10^16^
Multilevel Switching	Yes	Yes	No	Yes	No
Throughput (Mbits/s)	100	50	100	200	72
Energy Efficiency (nJ/bit)	0.1 ~ 2.5	2 ~ 25	0.37 × 10^−3^	2.7	389
Reference	[182,183]	[183,184]	[182,183,184]	[182,183,184]	[185]

* GPP stands for ‘General Purpose Processor’.
